# Correction: Dispersal of PRC1 condensates disrupts polycomb chromatin domains and loops

**DOI:** 10.26508/lsa.202302436

**Published:** 2023-10-23

**Authors:** Iain Williamson, Shelagh Boyle, Graeme R Grimes, Elias T Friman, Wendy A Bickmore

**Affiliations:** MRC Human Genetics Unit , Institute of Genetics and Cancer, University of Edinburgh, Edinburgh, UK

## Abstract

Endogenous PRC1–mediated chromatin compaction and clustering of polycomb target loci are reversibly perturbed in mESCs by the addition of 1,6 hexandiol.

Article: Williamson I, Boyle S, Grimes GR, Friman ET, Bickmore WA (2023 Jul 24) Dispersal of PRC1 condensates disrupts polycomb chromatin domains and loops. Life Sci Alliance 6(10): e202302101. doi: 10.26508/lsa.202302101. PMID: 37487640.

**Figure 1:**
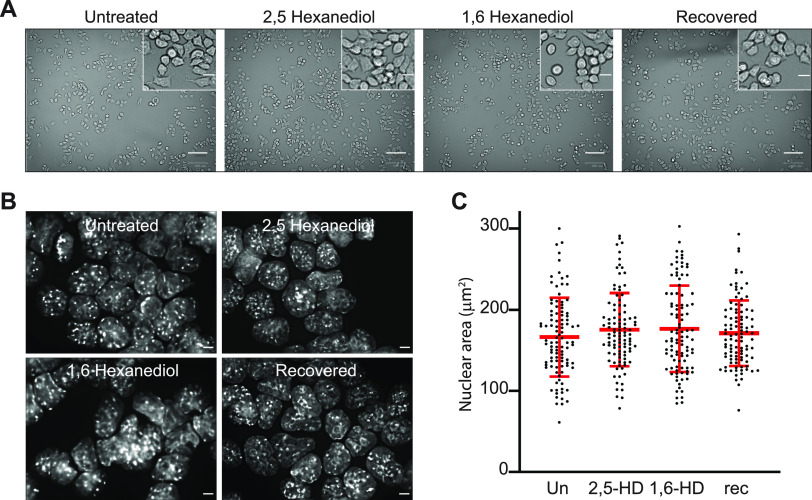


Correction to Figure 1: the authors replaced the zoomed image of the recovered cells in Figure 1A as the original image was the same as the zoomed image of untreated cells, that is, the same image (of untreated cells) was used for both.

