# Supplementary material for: Dispersal of PRC1 condensates disrupts polycomb chromatin domains and loops
Source: Life Sci Alliance. 2023 Jul 24;6(10):e202302101. doi: 10.26508/lsa.202302101 (PMC10366532; doi:10.26508/lsa.202302101)
Supplement: Supplementary file 1 [file LSA-2023-02101_TableS1.docx]

**Table S1. Effects of 2,5 or 1,6 hexanediol on mESC nuclear size.**

| **Treatment** | **Number of nuclei** | **Nuclear Area (μm^2^)** |
| --- | --- | --- |
| **Rep. 1**  **un**  **2,5-HD**  **1,6-HD**  **rec**  **Rep. 2**  **un**  **2,5-HD**  **1,6-HD**  **rec** | 100  100  100  100  100  100  110  120 | 166  176 (*p* = 0.17)  177 (*p* = 0.16)  171 (*p* = 0.46)  167  162 (*p* = 0.45)  159 (*p* = 0.18)  158 (*p* = 0.1) |

Related to Figs 1C and S1B. Nuclear area of untreated (un) mESCs and mESCs treated with either 2% 2,5 or 1,6 hexandiol (2,5-HD, 1,6-HD) for 5 minutes and for cells > 1-hour post-1,6-HD treatment (rec). Data are from two biological replicates (Rep.1 and Rep.2). Areas were measured by flattening (extended focus) the deconvolved 3D DAPI-stained images and calculating nuclear area using the freehand ROI tool on Volocity (see methods). Nuclear areas indicated (μm^2^) are mean values, statistical analysis by unpaired T Tests (two-tailed).
