# Supplementary material for: Dispersal of PRC1 condensates disrupts polycomb chromatin domains and loops
Source: Life Sci Alliance. 2023 Jul 24;6(10):e202302101. doi: 10.26508/lsa.202302101 (PMC10366532; doi:10.26508/lsa.202302101)
Supplement: Supplementary file 2 [file LSA-2023-02101_TableS2.docx]

**Table S2. Effects of 2,5 or 1,6 hexanediol on chromatin compaction at the HoxD and HoxB loci**

| Treatment | GCR-Lnp | Evx2-Hoxd3 | Evx2-Hoxd3  (Ring1B^-/-^) | Hoxb13-Hoxb1 |
| --- | --- | --- | --- | --- |
|  | **Interprobe distance (nm) and number of alleles [ ]** | | | |
| **Rep. 1**  **un**  **2,5-HD**  **1,6-HD**  **rec** | 329 [122]  334 (*p* = 0.83) [112]  288 (*p* = 0.19) [123]  287 (*p* = 0.11) [126] | 272 [92]  262 (*p* = 0.94) [100]  326 (*p* = 0.0099) [79]  268 (*p* = 0.88) [88] | 420 [82]  379 (*p* = 0.51) [81]  379 (*p* = 0.07) [60] | 494 [149]  515 (*p* = 0.038) [119]  496 (*p* = 0.9) [169] |
| **Rep. 2**  **un**  **2,5-HD**  **1,6-HD**  **rec** | 327 [100]  271 (*p* = 0.07) [100]  288 (*p* = 0.94) [90]  297 (*p* = 0.37) [86] | 307 [169]  286 (*p* = 0.62) [128]  333 (*p* = 0.019) [130]  323 (*p* = 0.76) [132] |  |  |

Statistical analysis of data for Fig.s 2C, E, G & Fig. S2A, B, C. Interprobe distances measured across the HoxB (Hoxb13-Hoxb1) and HoxD (Evx2-Hoxd3) loci and at a control locus adjacent to HoxD (GCR-Lnp) in two biological replicates of untreated (un) mESCs and in cells treated with 2% 2,5 or 1,6 hexanediol and for cells > 1-hour post-1,6-HD treatment (rec). Data from mESCs mutant for PRC1 (*Ring1B^-/^*^-^) are also shown. Interprobe distances shown are median values in nm. Square brackets indicate the number of alleles measured. Statistical analysis by Mann-Whitney U Tests.
