# Supplementary material for: Dispersal of PRC1 condensates disrupts polycomb chromatin domains and loops
Source: Life Sci Alliance. 2023 Jul 24;6(10):e202302101. doi: 10.26508/lsa.202302101 (PMC10366532; doi:10.26508/lsa.202302101)
Supplement: Supplementary file 3 [file LSA-2023-02101_TableS3.docx]

**Table S3. Effects of 2,5 or 1,6 hexanediol on clustering of *Irx3*, *Irx5*, and *Irx6***

| **Treatment** | **Wild type** | **Ring1B^-/-^** |
| --- | --- | --- |
|  | **Clustering (≤ 200 nm) frequency (%) of minimum of 2 of 3 loci and number of alleles [ ]** | |
| **Rep. 1**  **un**  **2,5-HD**  **1,6-HD**  **rec** | 23 [198]  25 (*p* = 0.69) [133]  15 (*p* = 0.019) [178]  30 (*p* = 0.19) [165] | 10 [108]  13 (*p* = 0.69) [136]  7 (*p* = 0.48) [126] |
| **Rep. 2**  **un**  **2,5-HD**  **1,6-HD**  **rec** | 26 [103]  21 (*p* = 0.35) [135]  8 (*p* = 0.002) [133]  21 (*p* = 0.34) [122] |  |
|  | **Dispersed (≥ 400 nm) frequency (%) of all 3 loci** | |
| **Rep. 1**  **un**  **2,5-HD**  **1,6-HD**  **rec** | 19  16 (*p* = 0.56)  38 (*p* = 0.0002)  14 (*p* = 0.33) | 30  35 (*p* = 0.41)  31 (*p* = 0.89) |
| **Rep. 2**  **un**  **2,5-HD**  **1,6-HD**  **rec** | 19  25 (*p* = 0.27)  37 (*p* = 0.02)  26 (*p* = 0.2) |  |

Statistical analysis of data for Figs. 3C, F; Fig. S3B. The proportion of alleles with clustering of *Irx3*, *Irx5*, and *Irx6* (≤ 200 nm between at least 2 of the three loci) or with dispersed alleles (≥ 400 nm between all 3 loci) in untreated (un) wild-type mESCs and in cells treated with 2% 2,5 or 1,6 hexanediol and for cells > 1-hour post-1,6-HD treatment (rec). Data from mESCs mutant for PRC1/Ring1B (R1B^-/-^) are also shown. *p*-values from Fisher’s Exact Tests. Data are from two independent biological replicates.
