# Supplementary material for: Dispersal of PRC1 condensates disrupts polycomb chromatin domains and loops
Source: Life Sci Alliance. 2023 Jul 24;6(10):e202302101. doi: 10.26508/lsa.202302101 (PMC10366532; doi:10.26508/lsa.202302101)
Supplement: Supplementary file 4 [file LSA-2023-02101_TableS4.docx]

**Table S4. The proportion of *En2*, *Shh*, and *Mnx1* clustering and dispersed alleles in un wild type and R1B^-/-^ mESCs compared to 2,5-HD, 1,6-HD and rec**

| **Treatment** | **Wild type mESCs** | **R1B^-/-^ mESCs** |
| --- | --- | --- |
|  | **Clustering (≤ 200 nm) frequency (%) of minimum of 2 polycomb targets and number of alleles [ ]** | |
| **Rep. 1**  **un**  **2,5-HD**  **1,6-HD**  **rec** | 34 [100]  29 (*p* = 0.46) [111]  13 (*p* = 0.0007) [100]  41 (*p* = 0.38) [100] | 7 [103]  13 [90]  11 [90] |
| **Rep. 2**  **un**  **2,5-HD**  **1,6-HD**  **rec** | 27 [100]  24 (*p* = 0.75) [100]  6 (*p* < 0.0001) [100]  20 (*p* = 0.32) [100] |  |
|  | **Dispersed (≥ 400 nm) frequency (%) of all 3 polycomb targets** | |
| **Rep. 1**  **un**  **2,5-HD**  **1,6-HD**  **rec** | 7  12 (*p* = 0.35)  32 (*p* < 0.0001)  10 (*p* = 0.61) | 46  34  41 |
| **Rep. 2**  **un**  **2,5HD**  **1,6HD**  **rec** | 19  17 (*p* = 0.85)  34 (*p* = 0.02)  24 (*p* = 0.49) |  |

Statistical analysis of data for Figs. 4C, F & S4A. *p*-values from Fisher’s Exact Tests.
