# Supplementary material for: Dispersal of PRC1 condensates disrupts polycomb chromatin domains and loops
Source: Life Sci Alliance. 2023 Jul 24;6(10):e202302101. doi: 10.26508/lsa.202302101 (PMC10366532; doi:10.26508/lsa.202302101)
Supplement: Supplementary file 5 [file LSA-2023-02101_TableS5.docx]

**Table S5. The proportion of *Cnpy1*, SFPE1, and *Ube3c* clustering and dispersed alleles in un wild type mESCs compared to 2,5-HD, 1,6-HD and rec**

| **Treatment** | **Wild type mESCs** |
| --- | --- |
|  | **Clustering (≤ 200 nm) frequency (%) of minimum of 2 non-polycomb targets and number of alleles [ ]** |
| **Rep. 1**  **un**  **2,5-HD**  **1,6-HD**  **rec** | 15 [150]  7 (*p* = 0.16) [60]  10 (*p* = 0.34) [100]  6 (*p* = 0.05) [84] |
| **Rep. 2**  **un**  **2,5-HD**  **1,6-HD**  **rec** | 4 [91]  10 (*p* = 0.14) [75]  7 (*p* = 0.71) [61]  10 (*p* = 0.2) [61] |
|  | **Dispersed (≥ 400 nm) frequency (%) of all 3 non-polycomb targets** |
| **Rep. 1**  **un**  **2,5-HD**  **1,6-HD**  **rec** | 32  42 (*p* = 0.2)  36 (*p* = 0.58)  27 (*p* = 0.55) |
| **Rep. 2**  **un**  **2,5-HD**  **1,6-HD**  **rec** | 43  39 (*p* = 0.64)  31 (*p* = 0.17)  28 (*p* = 0.09) |

Statistical analysis of data for Figs. 4E & S4B. *p*-values from Fisher’s Exact Tests.
