# Supplementary material for: Dispersal of PRC1 condensates disrupts polycomb chromatin domains and loops
Source: Life Sci Alliance. 2023 Jul 24;6(10):e202302101. doi: 10.26508/lsa.202302101 (PMC10366532; doi:10.26508/lsa.202302101)
Supplement: Supplementary file 6 [file LSA-2023-02101_TableS6.docx]

**Table S6. Comparison of the spatial distance between TAD boundary CTCF sites adjacent to *Shh* and ZRS in untreated mESCs with 2,5HD-treated, 1,6-HD-treated and recovered mESCs**

| **Treatment** | **Replicate 1** | **Replicate 2** |
| --- | --- | --- |
|  | **Interprobe distance (nm) and number of alleles [ ]** | |
| **un**  **2,5-HD**  **1,6-HD**  **rec** | 322 [67]  323 (*p* = 0.84) [86]  334 (*p* = 0.42) [80]  329 (*p* = 0.52) [81] | 322 [100]  282 (*p* = 0.65) [100]  278 (*p* = 0.68) [100]  298 (*p* = 0.86) [100] |

Statistical analysis of data for Fig. S4G. Interprobe distances are median values. *p*-values from Fisher’s Exact Tests.
