# Supplementary material for: Dispersal of PRC1 condensates disrupts polycomb chromatin domains and loops
Source: Life Sci Alliance. 2023 Jul 24;6(10):e202302101. doi: 10.26508/lsa.202302101 (PMC10366532; doi:10.26508/lsa.202302101)
Supplement: Supplementary file 7 [file LSA-2023-02101_TableS7.docx]

**Table S7. Comparison of the number of discrete polycomb and non-polycomb target foci in untreated mESCs with 2,5HD-treated, 1,6-HD-treated and recovered mESCs across chromosome 2**

| **Treatment** | **Polycomb +** | **Polycomb -** |
| --- | --- | --- |
|  | **Number of foci and number of alleles [ ]** | |
| **un**  **2,5-HD**  **1,6-HD**  **rec** | 15 [119]  16 (*p* = 0.02) [122]  16 (*p* = 0.001) [123]  13 (*p* = 0.047) [158] | 16 [124]  18 (*p* = 0.007) [125]  16 (*p* = 0.6) [127]  13 (*p* < 0.0001) [155] |

Statistical analysis of data for Fig. 5C. Foci numbers are median values, *p*-values from Mann-Whitney U Tests.
