# Supplementary material for: Dispersal of PRC1 condensates disrupts polycomb chromatin domains and loops
Source: Life Sci Alliance. 2023 Jul 24;6(10):e202302101. doi: 10.26508/lsa.202302101 (PMC10366532; doi:10.26508/lsa.202302101)
Supplement: Supplementary file 8 [file LSA-2023-02101_TableS8.docx]

**Table S8. Chr2 MyTags H3K27me3+ Regions**

| **Chromosome** | **Start** | **End** | **Ring1B peak** |
| --- | --- | --- | --- |
| chr2 | 42498000 | 42518000 | 1 |
| chr2 | 44380000 | 44400000 | 1 |
| chr2 | 49633000 | 49653000 | 1 |
| chr2 | 52521000 | 52541000 | 1 |
| chr2 | 54278000 | 54298000 | 0 |
| chr2 | 55278000 | 55298000 | 1 |
| chr2 | 56951000 | 56971000 | 1 |
| chr2 | 58200000 | 58220000 | 1 |
| chr2 | 60113000 | 60133000 | 1 |
| chr2 | 61640000 | 61660000 | 1 |
| chr2 | 65068000 | 65088000 | 0 |
| chr2 | 65952000 | 65972000 | 1 |
| chr2 | 68297000 | 68317000 | 1 |
| chr2 | 69410000 | 69430000 | 1 |
| chr2 | 70393000 | 70413000 | 1 |
| chr2 | 71365000 | 71385000 | 1 |
| chr2 | 73100000 | 73120000 | 1 |
| chr2 | 74560000 | 74580000 | 1 |
| chr2 | 76233000 | 76253000 | 1 |
| chr2 | 77644000 | 77664000 | 1 |
| chr2 | 79086000 | 79106000 | 0 |
| chr2 | 80276000 | 80296000 | 1 |
| chr2 | 81883000 | 81903000 | 1 |
| chr2 | 84705000 | 84725000 | 1 |
| chr2 | 90717000 | 90737000 | 1 |
| chr2 | 91754000 | 91774000 | 1 |
| chr2 | 92745000 | 92765000 | 0 |
| chr2 | 93785000 | 93805000 | 1 |

Mouse genome assembly number: NCBI m37. Probes previously used in (Boyle et al., 2020).
