# Supplementary material for: Dispersal of PRC1 condensates disrupts polycomb chromatin domains and loops
Source: Life Sci Alliance. 2023 Jul 24;6(10):e202302101. doi: 10.26508/lsa.202302101 (PMC10366532; doi:10.26508/lsa.202302101)
Supplement: Supplementary file 9 [file LSA-2023-02101_TableS9.docx]

**Table S9. Chr2 MyTags H3K27me3- Regions**

| **Chromosome** | **Start** | **End** |
| --- | --- | --- |
| chr2 | 43120000 | 43140000 |
| chr2 | 46675000 | 46695000 |
| chr2 | 51945000 | 51965000 |
| chr2 | 53038000 | 53058000 |
| chr2 | 56165000 | 56185000 |
| chr2 | 57571000 | 57591000 |
| chr2 | 59077000 | 59097000 |
| chr2 | 60673000 | 60693000 |
| chr2 | 63837000 | 63857000 |
| chr2 | 65285000 | 65305000 |
| chr2 | 67600000 | 67620000 |
| chr2 | 68800000 | 68820000 |
| chr2 | 69717000 | 69737000 |
| chr2 | 70862000 | 70882000 |
| chr2 | 72210000 | 72230000 |
| chr2 | 73524000 | 73544000 |
| chr2 | 75490000 | 75510000 |
| chr2 | 77056000 | 77076000 |
| chr2 | 78762000 | 78782000 |
| chr2 | 79510000 | 79530000 |
| chr2 | 81255000 | 81275000 |
| chr2 | 82760000 | 82780000 |
| chr2 | 84272000 | 84292000 |
| chr2 | 88200000 | 88220000 |
| chr2 | 91220000 | 91240000 |
| chr2 | 92090000 | 92110000 |
| chr2 | 93580000 | 93600000 |
| chr2 | 94233000 | 94253000 |

Mouse genome assembly number: NCBI m37. Probes previously used in (Boyle et al., 2020).
