# Supplementary material for: Dispersal of PRC1 condensates disrupts polycomb chromatin domains and loops
Source: Life Sci Alliance. 2023 Jul 24;6(10):e202302101. doi: 10.26508/lsa.202302101 (PMC10366532; doi:10.26508/lsa.202302101)
Supplement: Supplementary file 10 [file LSA-2023-02101_TableS10.docx]

**Table S10. Fosmid Probes**

|  | **Region** | **Whitehead (Sanger)**  **Name** | **Ensemble name** | **Coordinates**  Start End | **Size (bp)** |
| --- | --- | --- | --- | --- | --- |
|  |  |  |  |  |  |
| **Hoxd** | *GCR** | WI1-2157A11 | G135P63331H7 | 74242615 74282044 | 39429 |
|  | *Lnp** | WI1-482L15 | G135P61870C5 | 74329582 74372986 | 43404 |
|  | *Evx2-Hoxd13** | WI1-469P2 | G135P67444A12 | 74474157 74513003 | 38846 |
|  | *Hoxd4-Hoxd1** | WI1-121N10 | G135P67844B8 | 74566983 74605438 | 38455 |
| **Irx** | *Irx3*** | WI1-1206E19 |  | 94307305 94341470 | 34166 |
|  | *Irx5*** | WI1-1060D04 |  | 94865335 94904882 | 39548 |
|  | *Irx6*** | WI1-0901O22 |  | 95190971 95221152 | 30182 |
| **Shh** | *En2**** | WI1-2728F4 |  | 28477913 28517563 | 39650 |
|  | *Cnpy1**** | WI1-2816N11 |  | 28567538 28605641 | 38104 |
|  | *Shh**** | WI1-574O18 | G135P64333A4 | 28754458 28795879 | 41421 |
|  | *SFPE1**** | WI1-0552F05 |  | 28798551 28841224 | 42673 |
|  | *ZRS***** | WI1-1047E14 | G135P600929F6 | 29611727 29653695 | 41968 |
|  | *Mnx1**** | WI1-1204B6 |  | 29791124 29827491 | 36368 |
|  | *Ube3c**** | WI1-0492O10 |  | 29873876 29916051 | 42186 |

Names are Ensembl (r 45) (http://jun2007.archive.ensembl.org/Mus_musculus/index.html). Mouse genome assembly number: NCBI m37. Asterisks indicate fosmids previously used in: * (Williamson et al., 2012) ** (Sobreira et al., 2021) *** (Boyle et al., 2020) **** (Williamson et al., 2016, 2019)
