# Supplementary material for: Dispersal of PRC1 condensates disrupts polycomb chromatin domains and loops
Source: Life Sci Alliance. 2023 Jul 24;6(10):e202302101. doi: 10.26508/lsa.202302101 (PMC10366532; doi:10.26508/lsa.202302101)
Supplement: Supplementary file 11 [file LSA-2023-02101_TableS11.docx]

**Table S11. qPCR primers for ChIP analysis**

| **Promoter/exon** | **Oligo name** | **Sequence** |
| --- | --- | --- |
| *Hoxd10* | Hoxd10prof | TAGTAGATGTCGCTGTTGTCCG |
|  | Hoxd10pror | ACATGACAACCAAGCCAATGAGA |
| *En2* | En2intronf | CAACTCTGGGTGCTCTCCTG |
|  | En2intronr | GCTTGCAGGATGGAACGAAC |
| *Actin* | Actinf | CCTCGATGCTGACCCTCATCC |
|  | Actinr | GACACTGCCCCATTCAATGTCTC |
